# Prognostic Communication at the Limits of Viability: A Retrospective Mixed-Methods Study

**DOI:** 10.3390/children13101305

**Published:** 2026-09-25

**Authors:** Ephraim Friedman, Michal Bertele, Noga Avni Slutzky, Jacky Herzlich

**Affiliations:** 1 Department of Neonatology, Dana-Dwek Children’s Hospital, Tel Aviv Medical Center, Tel Aviv 6423906, Israel; michalber@tlvmc.gov.il (M.B.); jackyh@tlvmc.gov.il (J.H.); 2 School of Social Work, Bar-Ilan University, Ramat Gan 5290002, Israel; 3 The Bob Shapell School of Social Work, Tel Aviv University, Tel Aviv 6423906, Israel; nogav@tlvmc.gov.il; 4 Gray Faculty of Medical and Health Sciences, Tel Aviv University, Tel Aviv 6423906, Israel

**Keywords:** prognostic communication, limits of viability, extremely preterm infants, neonatal intensive care, parents, quality of life, shared decision-making

## Abstract

**Highlights:**

**What are the main findings?**

- Prognostic communication in the context of extreme prematurity was often perceived by parents as predominantly risk-focused, with limited discussion of long-term survival, development, and quality of life.
- Parents’ later perceptions of their child’s quality of life did not always correspond to the severity of neonatal morbidity at discharge, highlighting a gap between early clinical prognostic assessments and lived experience.

**What are the implications of the main findings?**

- Clinicians should provide balanced prognostic communication that addresses not only risks and impairments but also possible survival, development, adaptation, and quality of life.
- Explicitly acknowledging uncertainty and the potential gap between clinical measures and future lived experience may help parents make informed decisions and develop more realistic expectations and hope.

**Abstract:**

**Background:** Although advances in neonatal care over the last two decades have improved survival among extremely preterm infants, neurodevelopmental disabilities remain a major concern with substantial uncertainty regarding long-term outcomes. Prognostic counselling at the limit of viability is therefore challenging, with divergent perspectives between parents and clinicians further contributing to this challenge. **Objective:** To examine parents’ retrospective interpretations of prognostic communication in the context of extreme prematurity and their experiences of long-term adaptation and perceived child well-being. **Methods:** This retrospective mixed-methods study interviewed parents of infants born at 23–25 weeks’ gestation, 5–10 years after discharge or death. Qualitative data were analysed using Braun and Clarke’s six-phase thematic approach and integrated with neonatal clinical data collected from medical records. **Results:** Fourteen parents participated. Five interrelated themes were identified: forged by adversity, enduring emotional toll, vertigo and decisional overload, limits of prognostic communication and long-term impact. Five of ten parents of surviving infants reported that early discussions were predominantly risk-focused and insufficiently addressed uncertainty or potential positive trajectories. Among seven infants classified as having severe neonatal morbidity at discharge, five were rated by their parents as having good or excellent current overall well-being at the time of interview. Among bereaved parents, three of four described meaning-making and personal transformation reflected in the theme ‘Forged by adversity’; these experiences may be consistent with aspects of post-traumatic growth, although post-traumatic growth was not formally assessed. **Conclusions:** Some parents perceived risk-focused prognostic communication as shaping aspects of their subsequent experience. Counselling that transparently presents risks while explicitly acknowledging uncertainty and developmental variability may better support families beyond the neonatal period.

## 1. Introduction

Extremely preterm infants (23^0^⁄_7_ to 25^6^⁄_7_ weeks’ gestation) represent one of the most challenging situations in neonatal care [1]. Over the last 20 years, alongside evolving care practices, survival outcomes among preterm infants have improved, with increasing survival to discharge and survival without major neonatal morbidity [2]. However, neurodevelopmental disabilities continue to affect a substantial proportion of children born preterm, with increasing rates as gestational age at birth decreases [3]. Consequently, families face the ongoing burden of prolonged hospitalisation, prognostic uncertainty and high-stakes decisions with long-term implications [4].

Historically, research and clinical follow-up of children born preterm have focused predominantly on neurodevelopmental outcomes, and consequently, the concepts and definitions used to characterize long-term outcomes have largely evolved within this framework. These outcomes have served not only as measures of long-term morbidity, but also as the primary framework through which prognosis has been communicated and discussed with families, shaping how clinicians convey expectations and risks and how conversations surrounding decision-making and long-term care are approached [5]. However, as parental perspectives are typically not incorporated, these outcomes may not reflect what is most meaningful to families [5]. Behaviour, for example, is one of the factors parents most frequently prioritise, yet it is not typically included among the neurodevelopmental outcomes. This may partly explain why parental ratings of their child’s health do not always align with physician-assessed severity, particularly among children with severe outcomes [5,6]. Differences between parents’ and clinicians’ perspectives extend beyond severity classification to the interpretation of survival and disability outcomes during prognostic discussions [7]. These divergent perspectives may contribute to gaps in communication between clinicians and parents, with potential implications for high-stakes decision-making [5]. Previous research has emphasized the need for more comprehensive and balanced definitions of outcomes that incorporate aspects considered meaningful by parents [3,5,6,8]. Given the complexity of prognostic discussions, counselling should be approached as a bidirectional, collaborative, and ongoing process [7].

This study explores parents’ retrospective experiences resulting from prognostic communication following extremely preterm birth and examines how these experiences relate to long-term adaptation and perceptions of their child’s outcome.

## 2. Methods

### 2.1. Study Design

This mixed-methods study integrated quantitative clinical data with qualitative thematic analysis.

### 2.2. Setting

The study was conducted in the neonatal intensive care unit (NICU) of Dana-Dwek Children’s Hospital, Sourasky Medical Center, Tel Aviv, Israel, a tertiary academic medical centre providing neonatal intensive care to a diverse urban population and serving as a referral centre for high-risk pregnancies and extremely preterm infants. Interviews were conducted between 22 June 2025 and 4 July 2025.

### 2.3. Participants

Parents of infants born at 23^0^⁄_7_–25^6^⁄_7_ weeks’ gestation between 1 January 2013 and 31 December 2022 were eligible for study inclusion. Parents of infants who died within the first five days of life were excluded, as this population was considered distinct and beyond the scope of the present study.

### 2.4. Data Collection

Clinical and demographic data were extracted from electronic medical records and included gestational age, birth weight, duration of NICU stay, survival status and major complications. Structured telephone interviews were conducted using a predefined guide (Appendix A) that addressed communication with clinicians, emotional responses, involvement in decision-making, reflections after discharge or loss, and current overall well-being, using a non-validated five-category scale ranging from “excellent” to “poor”.

### 2.5. Data Analysis

A qualitative analysis was conducted according to Braun and Clarke’s six-phase thematic analysis [9]. The principal investigator (EF), a paediatrician with neonatal clinical experience, conducted all interviews and was not involved in the clinical care of any of the infants included in the study. All interviews were conducted in Hebrew, audio-recorded, and subsequently transcribed and translated into English by the PI. Interviews lasted approximately 30–45 min and followed a structured interview guide comprising the same core questions for all participants, while allowing participants to elaborate freely on their experiences. For bereaved parents, questions that were not relevant were omitted, while the core domains of the interview guide were retained. All parents were informed that they could pause or stop the interview at any time. Emotional support from the NICU social work team was available if needed; however, no parent requested such support.

The PI conducted all inductive coding and iterative theme development. No second researcher independently coded the data, and no formal inter-rater reliability or consensus coding process was undertaken. Throughout the interview and analytic processes, the PI remained reflexively aware that clinical experience in neonatology, as well as personal, family, cultural, and religious background, could have influenced the interview process, coding, and interpretation of participants’ narratives. Transcripts were not returned to participants for comment. Reporting followed the COREQ checklist.

The research team classified infants according to diagnoses documented in the neonatal discharge summary that were considered potentially relevant to long-term developmental outcome. Severe neonatal morbidity was defined as the presence of one or more of the following: grade III–IV intraventricular haemorrhage, periventricular leukomalacia, stroke, disseminated intravascular coagulation, or necrotizing enterocolitis. Infants who survived to discharge but did not meet any of these criteria were classified as having moderate neonatal morbidity.

The study used a retrospective mixed-methods design in which descriptive quantitative data from the medical records were used to characterize the clinical course and provide context for the qualitative findings. Integration occurred at the interpretive level by examining qualitative themes in relation to selected clinical characteristics and outcomes. Given the small sample size, no inferential statistical analyses were performed.

### 2.6. Ethics

Our institutional review board, the “Helsinki Committee”, approved the study (0332-23-TLV, 29 August 2023). All participants verbally provided informed consent.

## 3. Results

Forty-six infants met the inclusion criteria. Of eligible parents, twelve could not be contacted, seven were excluded due to infant death within five days of birth, and thirteen declined participation, resulting in a final sample of 14 parents (Figure 1). Four infants (28.6%) died during hospitalization, and ten (71.4%) survived to discharge. The median gestational age of the 14 infants was 24 weeks and 6 days (IQR 24 + 3 to 25 + 3), their median birth weight was 705 g (IQR 645–766) and their median NICU stay was 116.5 days (IQR 50–137). Twelve parents were aged 30–49 years and two were ≥50. Most parents were married and university-educated, and most resided in central regions (Table 1).

Thematic analysis identified five themes reflecting parents’ experiences across the period from their infant’s NICU admission through to long-term reflection (Table 2). Themes ranged from the emotional toll to the coping strategies parents developed, practical and relational changes in their lives, and their long-term reflections.

1. Forged by adversity. Nine parents described extreme prematurity as transformative. Despite fear and uncertainty, they reported that it led to personal growth and redefined priorities.
2. Enduring emotional toll. Eight parents described emotional distress and a lasting emotional toll, including anxiety, hypervigilance, and disrupted expectations of parenthood.
3. Vertigo and decisional overload. Five parents described rapidly evolving information, complex terminology and repeated high-stakes discussions as contributing to cognitive and emotional disorientation.
4. Limits of prognostic communication. Eight parents described limited understanding and involvement in decision-making, feelings of helplessness, and prognostic discussions focused predominantly on worst-case scenarios, with limited acknowledgment of uncertainty or positive trajectories.
5. Long-term impact. Five parents described feeling insufficiently prepared for life after NICU discharge. They reported that the ongoing demands of caring for a child born extremely preterm, along with uncertainty regarding future development, became central aspects of their lived experience.

Further examination of thematic narratives in relation to clinical course revealed patterns within specific themes across survivor and bereaved subgroups. ‘Long-term impact’ was evident in reports from five of the ten parents of surviving infants. These parents described receiving insufficient information regarding long-term survival, development, and expected quality of life. Additionally, among the ten surviving infants, seven were classified as having severe neonatal complications. Among the seven infants classified as having severe neonatal morbidity at discharge, three had grade III intraventricular hemorrhage, one had necrotizing enterocolitis, one had periventricular leukomalacia, and two had a stroke. Of these seven infants, five were rated by their parents as having good or excellent current well-being and quality of life. Among the parents of these five infants, four described experiences consistent with the theme of ‘Limits of prognostic communication’.

‘Forged by adversity was’ reflected among six of eight married parents of surviving infants. These six parents had additional children later. Similarly, among bereaved parents, three of four described meaning-making and personal transformation reflected in the theme ‘Forged by adversity,’ and two later chose to have additional children. ‘Enduring emotional toll’ was expressed by two of eight married parents of surviving infants who did not have additional children, reflecting a persistent sense of crisis and emotional burden. Three parents described significant long-term emotional or practical burdens. One parent of an infant who survived described persistent distress, including sensory triggers and intense emotional reactions that may be consistent with post-traumatic distress. She stated that ‘the beeps in McDonald’s take me straight back to the NICU.’ She also described repeated discussions about resuscitation in the delivery room and NICU as emotionally overwhelming and insensitive: ‘Every day they asked whether to save our baby … we were new parents … it was insensitive.’ She subsequently divorced. This individual narrative illustrates the emotional burden described by some parents but does not establish a relationship between neonatal communication and marital dissolution.

## 4. Discussion

### 4.1. Clinical Morbidity and Parental Perceptions of Well-Being

This retrospective mixed-methods study examined how parents of extremely preterm infants retrospectively interpreted prognostic communication and described their long-term experiences of survival, disability, loss, and family life. The findings revealed a consistent communication gap between parents and physicians in the context of extreme prematurity. Parents described communication that emphasized risks while providing limited information about long-term survival, development, and expected quality of life. Participants generally emphasized the importance of balanced and honest information that would allow emotional and practical preparation for life beyond the NICU.

A related finding was a discordance between discharge severity classifications and parents’ ratings of their children’s current overall well-being. This phenomenon has been described in the broader literature as the “disability paradox,” referring to satisfactory or positive quality of life despite clinically defined disability or impairment [10]. Our findings should not be interpreted as suggesting that early clinical assessments were inaccurate or that discharge morbidity classifications fail to predict long-term outcomes. Rather, they highlight that clinical classifications and parent-reported well-being capture different dimensions of outcome. Parents may place greater value on daily functioning, relationships, behaviour, independence, and family life, which may not be fully reflected in clinical morbidity classifications [11,12,13,14,15,16]. In our cohort, five of seven survivors classified as having severe neonatal morbidity at discharge were reported by their parents to have good or excellent current overall well-being. Four of these five parents also described experiences consistent with the theme “Limits of prognostic communication.” This distinction is relevant to prognostic communication, as parents may interpret the implications of extreme prematurity through the lens of hope, personal values, and everyday functioning, even in the presence of long-term health concerns [11,12,13,14,15,16].

### 4.2. Post-Traumatic Growth and Emotional Burden

The theme “Forged by adversity” may also be viewed through the conceptual framework of post-traumatic growth, which describes positive psychological changes that may emerge following profound adversity [17]. In our study, three of four bereaved parents described meaning-making and personal transformation following their experience of extreme prematurity and loss. These accounts may be consistent with aspects of post-traumatic growth, but the construct was not formally assessed and should therefore not be considered a study outcome. Given the small number of bereaved parents, this interpretation remains tentative. Previous research highlights the importance of supporting parental participation in decision-making, showing that discussions of treatment options may enhance parents’ perceived involvement and strengthen trust in physicians [15]. Nevertheless, decision-making surrounding extreme prematurity may also impose a substantial emotional burden, particularly when parents are asked to engage repeatedly with high-stakes decisions during an already overwhelming period. This was illustrated by a parent in our study who described repeated discussions regarding resuscitation as profoundly distressing and emotionally overwhelming. Regardless of its potential benefits and burdens, shared decision-making in cases of extreme prematurity may not always be feasible given the high stakes and narrow timeframe, and clinical decisions may therefore necessarily rely more heavily on prognostic information [11,12]. The emotional burden was also reflected in parents’ accounts of neonatal loss, ongoing impairment, and changes in family and marital life. Conversely, some parents reported having additional children after the neonatal experience, illustrating the diverse ways in which parents subsequently navigated their lives and family-building experiences.

### 4.3. Implications for Prognostic Communication

Clinically, these findings support the need for prognostic communication in cases of extreme prematurity to extend beyond risk stratification. While transparent discussion of mortality and morbidity is essential, neonatal counselling may benefit from structured communication of uncertainty and possible survivorship trajectories alongside risk disclosure. Involving a social worker in prognostic discussions may help elicit parents’ concerns and informational needs, support complex decision-making, and facilitate connections with relevant community-based services after discharge. More broadly, acknowledging developmental variability and possible positive trajectories may help parents develop a balanced understanding of their child’s future. This approach does not imply reassurance or prediction of a favourable outcome, rather it communicates the substantial risks of extreme prematurity while acknowledging uncertainty and individual variability, thereby supporting realistic hope without minimizing potential adverse outcomes. These findings also highlight the need for broader perspectives in neonatal research, including outcomes that reflect the experiences of survivors and their parents. Although neurodevelopmental impairment may impose substantial emotional and financial burdens, its longer-term effects on family adaptation and quality of life remain uncertain [18]. Greater involvement of parents in outcome selection and prioritization of outcomes that matter most to parents and other stakeholders should therefore be considered in future neonatal research [16].

## 5. Limitations

This single-centre study had a small and relatively homogeneous sample, limiting generalizability. Only 14 of 46 eligible infants were represented, introducing potential selection and participation bias. The retrospective design may also have introduced recall bias, as parental perceptions of prognostic communication may have been influenced by subsequent outcomes. Thus, associations with later parental experiences should not be interpreted as causal. Findings may also have limited transferability across different cultural, socioeconomic, and healthcare contexts. Finally, excluding infants who died within five days limits applicability to families experiencing early neonatal loss.

## 6. Conclusions

These exploratory findings suggest that some parents retrospectively perceived risk-focused prognostic communication as shaping aspects of their subsequent experience. Counselling that clearly presents risks while acknowledging uncertainty and potential variability in developmental trajectories may better address parents’ informational and emotional needs during and beyond the NICU course.

## Figures and Tables

**Figure 1:**
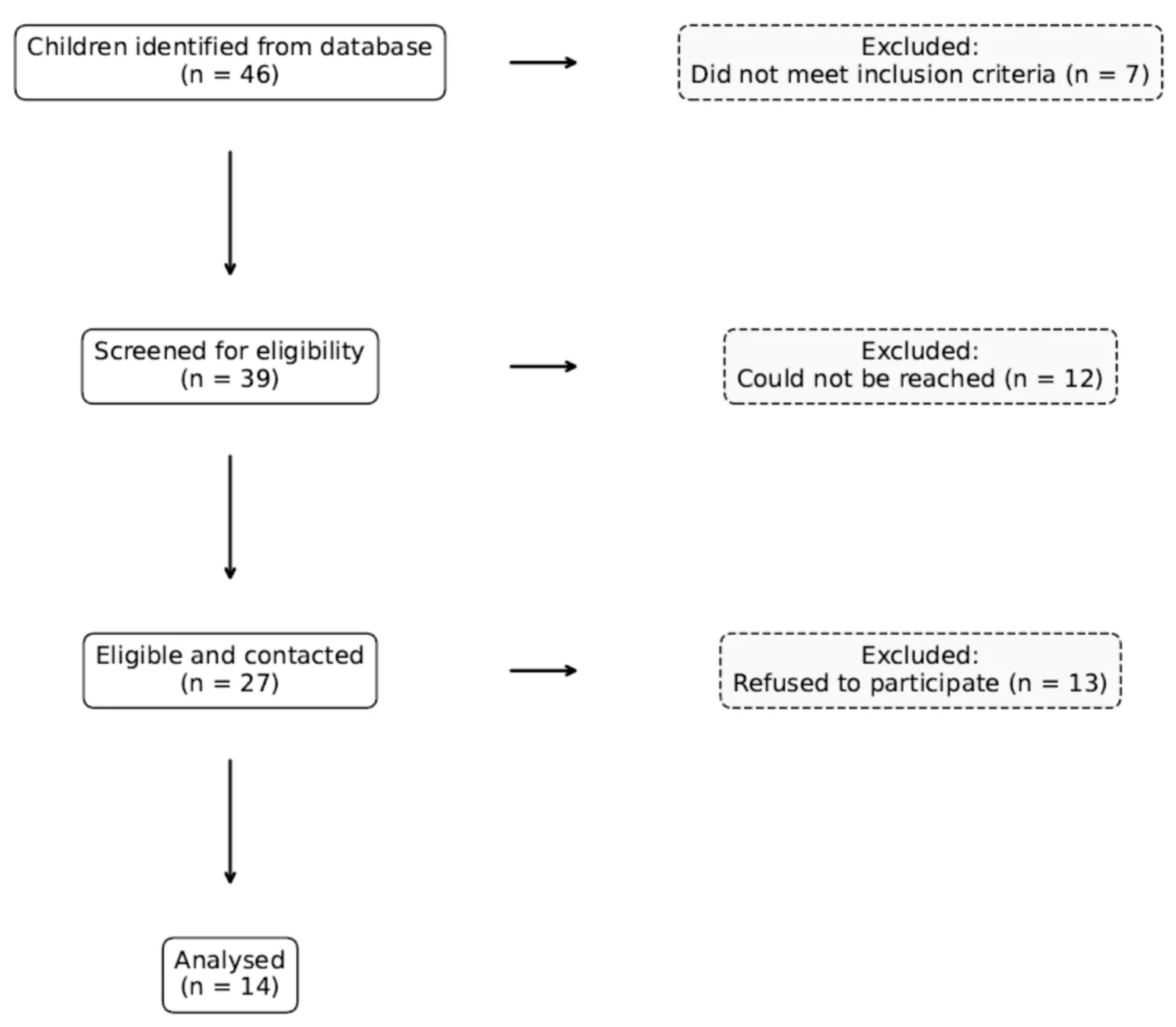
Flow diagram of study participants.

**Table 1:** Participant characteristics.

**Characteristic of Infants**	**Value**
Sex	
Male	8 (57.1)
Female	6 (42.9)
Severe complications	7 (50.0)
Moderate complications	3 (21.4)
Died in NICU	4 (28.6)
Gestational age at birth, n (%)	
Week 23	0 (0%)
Week 24	7 (50.0%)
Week 25	7 (50.0%)
Survived to hospital discharge (n = 10)	
Gestational age at birth, weeks	24 + 4 (24 + 3–25 + 6)
Birth weight, grams	680 (500–1085)
Length of NICU stay, days	123 (50–174)
Died during hospitalization (n = 4)	
Gestational age at birth, weeks	25 + 3 (24 + 6–25 + 6)
Birth weight, grams	798.5 (400–887)
Length of NICU stay, days	13 (5–51)
**Characteristic of Parent**	**Value**
Sex	
Male	2 (14.3)
Female	12 (85.7)
Age group, years	
30–39	7 (50.0)
40–49	5 (35.7)
≥50	2 (14.3)
Highest level of education	
Academic	11 (78.6)
High school	3 (21.4)
Religious practice level	
Secular	11 (78.6)
Traditional	2 (14.3)
Religious	1 (7.1)
Marital status	
Married	10 (71.4)
Single parent	2 (14.3)
Divorced	1 (7.1)
Partnered	1 (7.1)
Additional children *	8 (57.1)
Region of residence	
Central region	12 (85.7)
Northern region	1 (7.1)
East region	1 (7.1)
Years since birth of infant	
5	3 (21.4)
6	5 (35.7)
7	2 (14.3)
8	1 (7.1)
10	2 (14.3)
>10	1 (7.1)

Categorical variables presented as n (%); continuous variables presented as median (IQR); * born after NICU experience.

**Table 2:** Parent replies by theme.

Theme	Parent ID	Question No	Quote
Forged by adversity	7	16	“We could have given up, on the other hand, it brought us to where we are, the whole time the song playing in my mind was ‘at the end of the road you will be happy.”
	7	17	“Even though she did not survive, it was meant to happen, it changed things in our lives. We accumulated emotional residue from the first preterm baby, and only after she died we went to therapy and processed the experiences. It was meaningful.”
	8	16	“It is hard and complicated, but in the end we managed, and we were rebuilt from it.”
	16	16	“Our whole lives were shaped by this … we appreciate life more.”
	22	16	“It changed my personality, different proportions, it taught us about our marital and family structure. The endurance you recruit is what you have. We learned to look the truth in the face and cope with it.”
	30	16	“I learned to be less perfectionistic, I resigned from my job in order to be at home, it helped me to develop and to find something better later.”
	41	16	“I learned many things about myself and my husband, we acted as a team, it was challenging, but we succeeded. It gave us new proportions for the world, what is important and what is less.”
Enduring emotional toll	36	16	“It affected my daughter, our relationship, our entire family, it affects you physically and emotionally, crying all day … always worrying.”
	36	16	“It was a very difficult period, spending every day in the hospital and afterwards coping with the epilepsy.”
	36	16	“This is not what I imagined when I got married.”
	39	16	“The birth changed everything … being discharged with oxygen and a monitor is not a normal motherhood experience. It was a shocking experience, not something you prepare yourself for.”
	38	16	“It affected every part of life harming our relationship, quality of life, my abilities, my career, and my aspirations.”
	35	18	“A very difficult period … an emotional rollercoaster.”
	30	22	“It was an insane trauma, but all’s well that ends well.”
	41	16	“It is the most traumatic and the happiest thing that I have gone through.”
Vertigo and decisional overload	22	18	“People who are inside this catastrophe have no mental space, they cannot listen.”
	35	17	“Even after a week you do not understand what is going on.”
	36	19	“It is very difficult to see that they let children go. How is there an option of not doing everything?!”
	38	22	“In the prenatal period every day they asked whether to save our baby … we were new parents … it was insensitive.”
	38	22	“When parents come with their grown children to visit the NICU, it is nice, but I am fighting for my child’s life, it does not give me hope, it pinches the heart.”
	41	22	“If I had known in advance her developmental chances, I would have asked not to perform resuscitation, thanks to my ignorance today she is my miracle.”
Limits of prognostic communication	35	17	“In the first days everything was shown to us in a gloomy light … everything is bad … don’t panic, listen to them and also don’t listen.”
	16	16	“We learned to check things thoroughly, and not to rely on the rabbi or the doctor, and it made us resilient.”
	16	17	“Be there as much as possible, to be involved in all the processes and decisions.”
	16	19	“We chose to decide wherever we were able to.”
	8	21	“Sometimes they are too frightening, they’re not sensitive enough. They need to give at least a little support, not everything is bad, some children manage.”
	22	21	“They didn’t explain what was happening, we weren’t included in decision-making … we felt disconnected.”
	27	21	“I had no control … not on the treatment, not over my baby or anything that was happening there.”
	38	17	“Listen only to yourself, don’t do what the doctors say. Always keep your eyes open … there were cases where our presence changed a lot. Be involved.”
	38	17	“Don’t automatically trust the system, be on top of things.”
	38	18	“Trust the doctors but trust yourself more.”
	39	21	“I did not know what was happening with him, what they are doing, what the risks are.”
	36	21	“They were very pessimistic, they didn’t give even a drop of hope … staff needs to be careful, parents are in a complex emotional state.”
Long-term impact	22	22	“No one talks about the day after the NICU, things need to be said clearly and explicitly.”
	30	21	“We want to receive clear answers, but no one knows.”
	35	16	“While getting organized to leave the house, he has incontinence in the car. At the mall he runs away from me because he does not understand.”
	35	21	“They did not speak in simple terms. They told the truth, but the bottom line was missing.”
	36	16	“It’s not easy to this day, and it won’t be in the future … it’s very intensive; we try to hold on to the good things and focus on the positive.”
	36	16	“Ultimately, you want him to be able to manage on his own.”
	36	21	“I would have liked to hear about a range of complications that can happen, to hear in detail what life with her would be like, how to treat, what to prepare for.”
	30	16	“I was always a person who planned ahead, but I understood that it is impossible to plan now, I need to live, live with loss of control.”

## Data Availability

The data presented in this study are available on request from the corresponding author, the data are not publicly available due to privacy and confidentiality considerations regarding the study participants.

## Appendix A. Structured Interview Guide

Section A: Demographic and Background Information

1. Study ID number (assigned by the research team)
2. Age (years)
3. Sex
4. Area of residence
5. Level of education (primary, secondary, academic)
6. Religion (Jewish, Christian, Muslim, Druze, other)
7. Religious affiliation/practice level (religious, traditional, secular)
8. Current marital status (single parent, partnered, married, divorced)
9. Marital status at the time of birth (single parent, partnered, married, divorced)
10. How many children do you have?
11. How many children did you have prior to the preterm infant?

Section B: Knowledge and Perceptions of Prematurity

12. Prior to delivery, how much did you know about prematurity and its possible complications compared to your current level of knowledge?
  (1–5 scale; 1 = very little, 5 = a great deal)
13. During the first 48 h in the NICU, how much did you know about prematurity and its possible complications compared to your current level of knowledge?
  (1–5 scale; 1 = very little, 5 = a great deal)
14. At the time of discharge from the NICU, how much did you know about prematurity and its possible complications compared to your current level of knowledge?
  (1–5 scale; 1 = very little, 5 = a great deal)
15. Which person was most significant to you during the most challenging hours in the NICU? (parents, siblings, neonatologist, social worker/psychologist, spiritual figure, friends, other; more than one answer may be selected)
16. How would you rate your child’s current overall well-being and quality of life?
  1. Excellent
  2. Good
  3. Fair
  4. Complex
  5. Poor

Section C: Open-Ended Questions

17. How did the birth affect your life?
18. If you could stand in the delivery room minutes before the birth and whisper something to yourself, what would you say?
19. If close friends were now facing the birth of a preterm infant at a similar gestational age to your child’s, what would you advise them?
20. How was the decision made regarding comfort care versus continuation of life-sustaining treatment?
21. What was helpful or positive about the neonatologists’ support and guidance?
22. What was lacking in the neonatologists’ support and guidance?
23. Additional comments
